# Cellulose-lanthanum hydroxide nanocomposite as a selective marker for detection of toxic copper

**DOI:** 10.1186/1556-276X-9-466

**Published:** 2014-09-03

**Authors:** Hadi M Marwani, Mazhar Ullah Lodhi, Sher Bahadar Khan, Abdullah M Asiri

**Affiliations:** 1 Department of Chemistry, Faculty of Science, King Abdulaziz University, P.O. Box 80203, Jeddah 21589, Saudi Arabia; 2 Center of Excellence for Advanced Materials Research (CEAMR), King Abdulaziz University, P.O. Box 80203, Jeddah 21589, Saudi Arabia

**Keywords:** Nanostructures, Nanocomposite, Cu(II), Modified cellulose phase, Separation, ICP-OES, Batch method

## Abstract

In this current report, a simple, reliable, and rapid method based on modifying the cellulose surface by doping it with different percentages of lanthanum hydroxide (i.e., 1% La(OH)_3_-cellulose (LC), 5% La(OH)_3_-cellulose (LC2), and 10% La(OH)_3_-cellulose (LC3)) was proposed as a selective marker for detection of copper (Cu(II)) in aqueous medium. Surface properties of the newly modified cellulose phases were confirmed by Fourier transform infrared spectroscopy, field emission scanning electron microscope, energy dispersive X-ray spectroscopy, X-ray diffraction, and X-ray photoelectron spectroscopic analysis. The effect of pH on the adsorption of modified cellulose phases for Cu(II) was evaluated, and LC3 was found to be the most selective for Cu(II) at pH 6.0. Other parameters, influencing the maximum uptake of Cu(II) on LC3, were also investigated for a deeper mechanistic understanding of the adsorption phenomena. Results showed that the adsorption capacity for Cu(II) was improved by 211% on the LC3 phase as compared to diethylaminoethyl cellulose phase after only 2 h contact time. Adsorption isotherm data established that the adsorption process nature was monolayer with a homogeneous adsorbent surface. Results displayed that the adsorption of Cu(II) onto the LC3 phase obeyed a pseudo-second-order kinetic model. Selectivity studies toward eight metal ions, i.e., Cd(II), Co(II), Cr(III), Cr(VI), Cu(II), Fe(III), Ni(II), and Zn(II), were further performed at the optimized pH value. Based on the selectivity study, it was found that Cu(II) is highly selective toward the LC3 phase. Moreover, the efficiency of the proposed method was supported by implementing it to real environmental water samples with adequate results.

## Background

Over the years, copper (Cu(II)) has gain the attention of chemists due to its prohibitive toxicity and nonbiodegradable nature. The elevated concentration of Cu(II) produces severe ecological and public health issues [1,2]. Several methodologies have been evaluated for the separation of Cu(II) in aqueous medium. However, the adsorption technique has proved to be one of the promising solutions due to its simple implementation and economical and effective behavior [3,4]. Currently, some of the available adsorbents have limitations, such as low uptake capacity, long equilibrium, and low selectivity [5]. In order to overcome these weaknesses, some new organic-inorganic hybrid adsorbents, capable of separating heavy metals from the solution, have been established. Several studies have been concentrated on the extraction of Cu(II) by applying amine group functionalized matrices [6-9]. It is well understood that organic functional group modified adsorbents usually exhibit relatively high adsorption capacity and selectivity as compared to unmodified adsorbents [6,10-12].

Different separation techniques, however, are being successfully utilized, for example, liquid-liquid extraction [13], ion exchange [14], coprecipitation [15], cloud point extraction [16], and solid phase extraction (SPE) [17,18]. The conventional methods, such as liquid-liquid extraction and coprecipitation, require excess amount of organic solvents with high purity that could be harmful to living organisms and cause environmental pollution. On the other hand, the SPE method proved to be a more efficient technique when it comes to the exposure and usage of solvents, extraction time, and disposal cost. Presently, this recognition of SPE leads to the appearance of several adsorbents with the goal of succeeding a selective separation of the analytes, for instance, alumina [19], C18 [20], molecular imprinted polymers [21], cellulose [22], silica gel [23], and activated carbon [24,25].

Cellulose is considered to be one of the highly abundant naturally existing polymers in the world. This comprises repeating units of β-d-glucopyranose, covalently linked with OH group of C4 and C1 carbon atoms [26-28]. Naturally occurring cellulose shows less adsorption capacity and physical stability due to the steric hindrance offered by three hydroxyl groups with the same ring. Moreover, these hydroxyl moieties are chemically unreactive as the polymer matrix contains crystalline regions [27,29]. In order to develop adsorption capacity and structural stability of natural cellulose, modifications were employed in the matrix by means of chemical reactions, such as halogenation, etherification, esterification, and oxidation. Such modified matrices were found to be capable of separating heavy metal ions from aqueous solutions [27]. The cellulose beads when treated mainly with 2-(diethylamino) ethyl chloride hydrochloride along with some other treatments produced diethylaminoethyl cellulose [30]. Recently, we have also developed some surface modified cellulose adsorbents for the selective separation of Ni(II) and Cr(VI) ions [31,32].

In order to monitor metal ionic species in the environment, the development of rapid, simple, and proficient approach has gain an interest. Different approaches were employed for the determination of metal ions in aqueous medium, namely atomic absorption spectrometry (AAS) [33], inductively coupled plasma-mass spectrometry (ICP-MS) and inductively coupled plasma-optical emission spectrometry (ICP-OES) [34-36], anodic stripping voltammetry [37], and ion chromatography [38]. Despite immense enhancement in selectivity and sensitivity of state-of-the-art instruments, there is still a vital need for improvement in selective separation of chemical species of interest prior to their determination; in particular, the concentration of such analytes is frequently low in complex matrices. Moreover, a cleanup step is frequently needed because of high level of other constituents accompanying the analyte.

Current study emphasizes the development of new cellulose-based adsorbents by surface modification. Lanthanum hydroxide was doped with cellulose with a proportion of 1%, 5%, and 10% [i.e., 1% La(OH)_3_-cellulose (LC), 5% La(OH)_3_-cellulose (LC2), and 10% La(OH)_3_-cellulose (LC3)]. Additionally, the effectiveness of nanocomposites was investigated as a potential adsorbent for a selective extraction of Cu(II) ion prior to its determination by ICP-OES. Several parameters were evaluated in order to acquire the optimum condition for Cu(II) extraction. The pH effect on Cu(II) adsorption was investigated and optimized for the best modified cellulose phase (LC3). In order to understand the mechanism of Cu(II) adsorption, other parameters controlling the maximum uptake of Cu(II) on LC3 was studied at the optimum pH 6.0. Furthermore, adsorption data was modeled by Freundlich and Langmuir adsorption isotherms. The kinetics of adsorption was evaluated by employing pseudo-first- and second-order kinetic models. At optimized pH, selectivity was also scrutinized for other metal ion, including Cd(II), Co(II), Cr(III), Cr(VI), Fe(III), Ni(II), and Zn(II). This study revealed that LC3 was the most selective toward Cu(II) in comparison to other metal ions. Ultimately, the proposed method was further validated by analysis of real environmental water samples.

## Methods

### Chemicals and reagents

Diethylaminoethyl (DEAE) cellulose, lanthanum chloride, and ethanol were purchased from Sigma-Aldrich (Milwaukee, WI, USA). Stock standard solutions of 1,000 mgL^-1^ Cd(II), Co(II), Cu(II),Cr(III), Cr(VI), Fe(III), Ni(II), and Zn(II) were obtained from Sigma-Aldrich (Milwaukee, WI, USA). All utilized reagents were of high purity and of analytical reagent grade, whereas double-distilled deionized water was used throughout the experiments.

### Preparation of the new solid phase extractor based on DEAE cellulose

Different amounts of DEAE cellulose (99%, 95%, and 90%) were first mixed with distilled deionized water. Various portions of lanthanum chloride (1%, 5%, and 10%) were then dissolved in distilled deionized water and mixed with DEAE cellulose water suspensions. All solutions were adjusted to pH 10.0 by a dropwise addition of 0.1-M NaOH. Mixtures were then allowed to stir at 60°C for 24 h. Mixtures were filtered, washed with ethanol twice and 18.2 MΩ cm distilled deionized water, and dried in oven at 80°C for 5 h to obtain LC, LC2, and LC3 nanocomposites.

### Samples preparation and procedure

Stock standard solutions of Cd(II), Co(II), Cu(II),Cr(III), Cr(VI), Fe(III), Ni(II), Pb(II), and Zn(II) ions were prepared in 18.2 MΩ cm distilled deionized water and stored in the refrigerator at 4°C. The environmental samples were collected from seawater, wastewater, tap water, and ground water from Jeddah region at Saudi Arabia.

#### ***Effect of pH***

The effect of pH on the adsorption of Cu(II) ion onto *n*% La(OH)_3_ DEAE cellulose was investigated. Standard solutions of 5.0 mgL^-1^ Cu(II) were adjusted to pH values ranging from 1.0 to 8.0 with appropriate buffer solutions, i.e., HCl/KCl buffer for pH 1.0 and 2.0, acetate buffer for pH 3.0 to 5.0, and KH_2_PO_4_/NaOH buffer for pH 6.0 to 8.0. Each solution was individually mixed with 25.0 mg of modified cellulose phases (LC, LC2, or LC3) and unmodified DEAE cellulose phase and mechanically shaken for 2 h by a mechanical shaker at 150 rpm and 25°C temperature.

#### ***Effect of concentration***

To estimate the uptake capacity of Cu(II) under batch conditions, standard solutions of 0, 1, 5, 10, 20, 30, 40, 50, 80, 100, 150, 200, 300, 400, and 500 mgL^-1^ were prepared and adjusted to the optimum pH 6.0 with the buffer solution of KH_2_PO_4_/NaOH. Each solution was individually added to 25.0 mg LC3 (or unmodified DEAE cellulose). All mixtures were then allowed to be mechanically shaken for 2 h at 25°C.

#### ***Effect of temperature***

For the effect of temperature, standard solutions of 5.0 mgL^-1^ Cu(II) were prepared, adjusted to the pH 6.0 as above, and individually mixed with 25.0 mg LC3. Thermodynamic study of the adsorption of LC3 toward Cu(II) was also performed under the same batch conditions at different temperatures (298, 308, 323, and 338 K).

#### ***Effect of shaking time***

The effect of shaking time on LC3 adsorption for Cu(II) was performed under the same batch conditions as above by given various equilibrium periods (5, 10, 20, 30, 50, 80, 100, and 120 min) and at a concentration of 400 mgL^-1^ of Cu(II).

#### ***Selectivity studies***

In order to investigate the selectivity of modified cellulose adsorbents toward different metal ions, including Cd(II), Co(II), Cu(II), Cr(III), Cr(VI), Fe(III), Ni(II), Pb(II), and Zn(II), 5 mgL^-1^ of each metal ion solution was individually added to 25.0 mg of modified phases ( LC, LC2, or LC3) and unmodified DEAE cellulose separately as well. Mixtures were then allowed to be stirred for 2 h at 25°C under the same batch conditions as above.

#### ***Apparatus***

The surface morphology of the nanocomposites was investigated by operating a field emission scanning electron microscope (FE-SEM) instrument (JSM-7600 F, JEOL Ltd., Akishima-shi, Japan). Elemental analysis was performed using energy dispersive X-ray spectroscopy (EDS) from JEOL, Japan. X-ray diffraction (XRD) patterns were acquired with X-ray diffractometer (Rigaku X-ray diffractometer, MiniFlex 2, Rigaku, Shibuya-ku, Japan) equipped with Cu-Kα1 radiation (*λ* = 1.5406 nm) using a generator voltage (40.0 kV) and a generator current (35.0 mA). Data of X-ray photoelectron spectroscopy (XPS) were acquired from Thermo Scientific K-α KA1066 spectrometer (Bonn, Germany)**.** The Al Kα X-ray radiation monochromatic sources were utilized as excitation sources, whereas the size of beam spot was set at 300.0 μm. The fixed analyzer transmission mode was used to record the spectra adjusting pass energy at 200 eV. The scan of spectra was performed at a pressure less than 10^-8^ Torr. Fourier transform infrared (FT-IR) spectroscopic analyses were carried out by using Shimadzu IR 470 spectrophotometer (Shimadzu, Kyoto, Japan) to confirm the formation of newly prepared nanocomposites. The pH measurements were performed on pH meter (inoLab® pH 7200, WTW, Lincolnwood, IL, USA) with absolute accuracy limits at the pH measurement being defined by NIST buffers. A PerkinElmer ICP-OES Optima 4100 DV model (PerkinElmer, Waltham, MA, USA) was applied for the determination of metal ions concentration. The optimization of ICP-OES instrument was performed daily before analysis and operated as recommended by the manufacturers.

The ICP-OES spectrometer was operated with the following parameters: FR power, 1,300 kW; frequency, 27.12 MHz; demountable quartz torch, Ar/Ar/Ar; plasma gas (Ar) flow, 15.0 L min^-1^; auxiliary gas (Ar) flow, 0.2 L min^-1^; nebulizer gas (Ar) flow, 0.8 L min^-1^; nebulizer pressure, 2.4 bar; glass spray chamber according to Scott (Ryton), sample pump flow rate, 1.5 mL min^-1^; integration time, 3 s; replicates, 3; wavelength range of monochromator, 165 to 460 nm. The concentrations of the metal ions were determined at wavelengths of 226.50 nm for Cd(II), 230.80 nm for Co(II), 267.72 nm for Cr(III and VI), 327.40 nm for Cu(II), 259.94 nm for Fe(III), 231.60 nm for Ni(II), and 202.55 nm for Zn(II).

## Results and discussion

### Characterization

**Figure 1:**
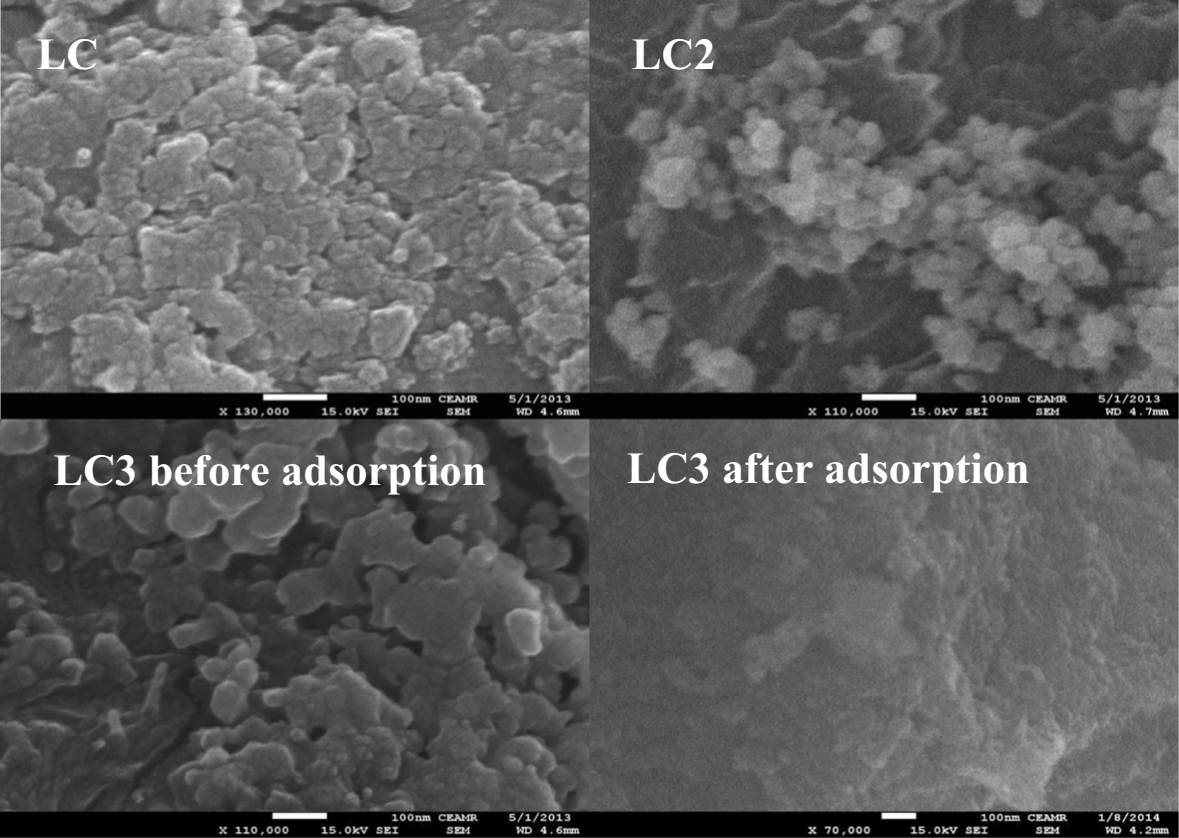
FE-SEM images of modified cellulose phases (LC, LC2, LC3) before adsorption and LC3 after adsorption.

**Figure 2:**
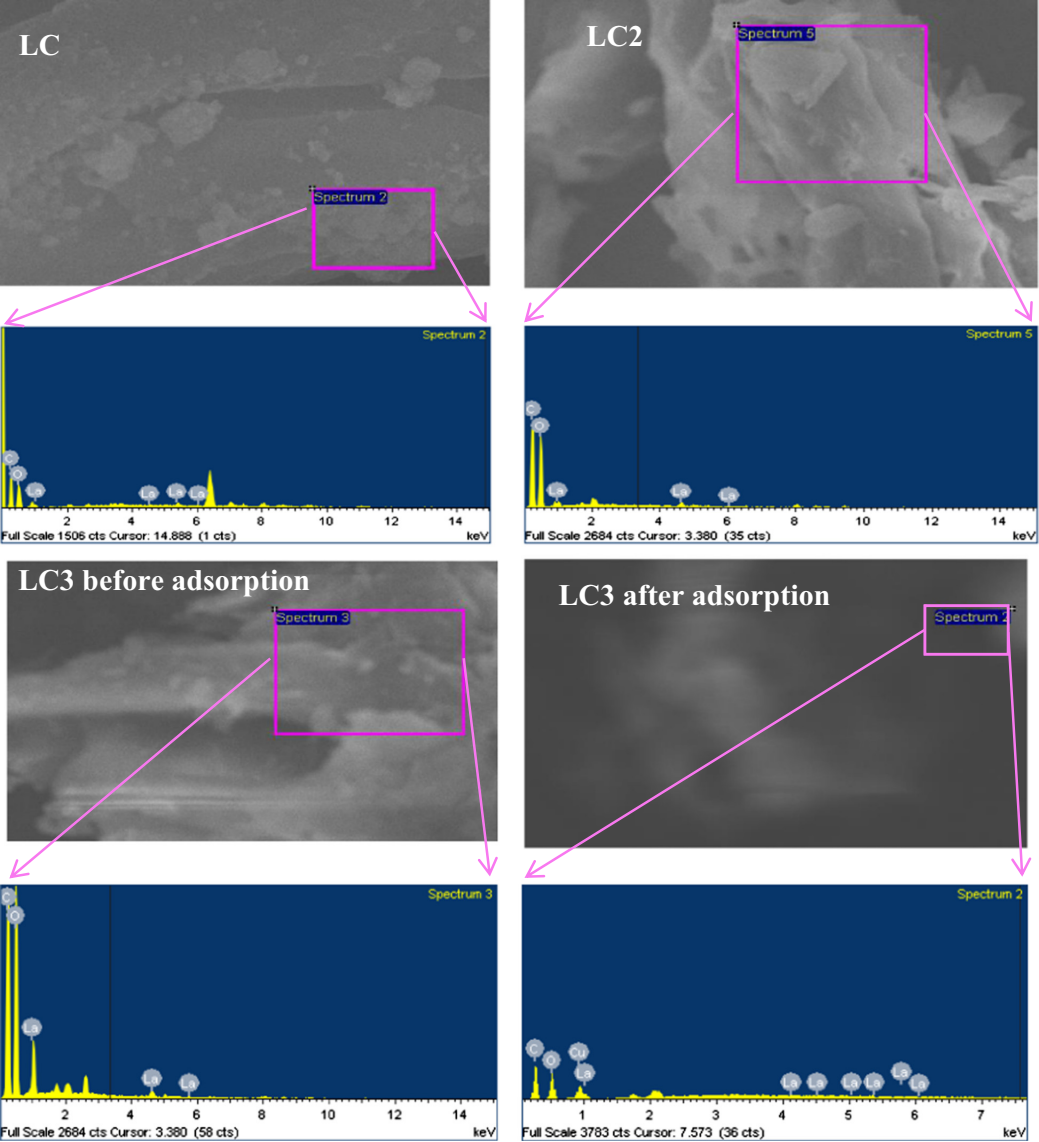
EDS spectra of modified cellulose phases (LC, LC2, LC3) before adsorption and LC3 after adsorption.

The morphological behavior of synthesized nanocomposites was studied by FE-SEM, as can be depicted in Figure 1. FE-SEM images of nanocomposites are comprised of aggregated nanoparticles with an average particle size of 50 nm. These aggregated nanoparticles are well distributed on the cellulose matrix, proposing that La(OH)_3_ nanoparticles are dispersed on the cellulose surface. Furthermore, it can be clearly observed from Figure 1 that the size of nanoparticles increases as the percentage of La(OH)_3_ doping increases due to aggregation of La(OH)_3_ on cellulose surface. Conversely, the surface of LC3 matrix was observed with finely adsorbed Cu(II) onto the cellulose surface after the adsorption process. The composition of these nanocomposites was evaluated by EDS spectrum, as shown in Figure 2. In EDS spectra of all modified phases, the peaks, related to carbon, oxygen, and lanthanum at 0.2, 0.5, and 1.0 keV, respectively, are observed without any other impurity peak. This indicates that the synthesized nanocomposites are composed of cellulose and La(OH)_3_. Adsorption of Cu(II) was also evaluated by EDS by attaining the spectrum of LC3 phase after adsorption. It is of interest to note that a peak at 0.9 keV for Cu(II) is observed in the LC3 spectrum after adsorption.

**Figure 3:**
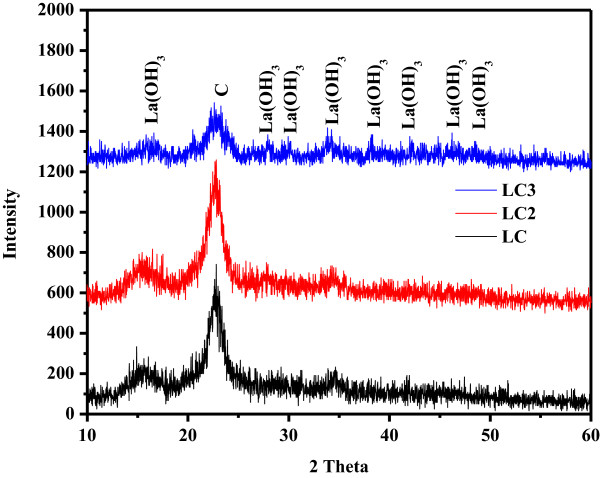
XRD spectra of modified cellulose phases (LC, LC2, and LC3).

The purity and crystallinity of synthesized nanocomposites were evaluated using XRD analysis, as displayed in Figure 3. In the XRD pattern of nanocomposites, a hallow peak at 22.0 Å is observed in all the modified cellulose phases (LC, LC2, and LC3), corresponding to amorphous nature of the adsorbents [39]. Moreover, XRD spectra also show several well crystalline peaks at 16.0, 28.0, 30.0, 34.0, 38.37, 42.31, 46.24, and 48.72 Å, describing the growth of La(OH)_3_ onto the surface of cellulose as reported in the literature [40]. Consequently, the diffraction peaks of all modified cellulose phases confirmed that synthesized products were nanocomposites of cellulose and La(OH)_3_ nanoparticles. Further, no peaks other than those for cellulose and La(OH)_3_ were observed in obtained diffraction patterns of modified phases, verifying that the synthesized nanocomposites are composed of cellulose and La(OH)_3_[40].

**Figure 4:**
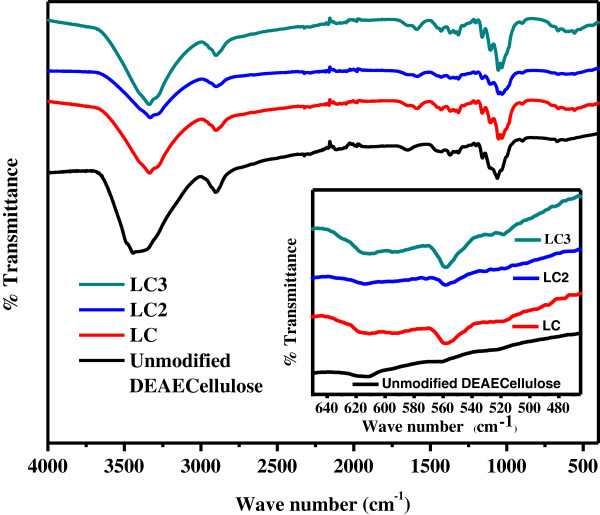
FT-IR spectra of modified cellulose adsorbents (LC, LC2, and LC3).

Chemical structures and functional groups presented in synthesized nanocomposites were evaluated by FT-IR spectra, as illustrated in Figure 4. FT-IR spectrum of unmodified DEAE cellulose exhibits a strong absorption band for OH stretching vibration at 3,450 cm^-1^ due to the presence of hydroxyl group. However, the same stretching band for hydroxyl group was shifted to 3,335 cm^-1^ in the spectra of modified cellulose phases (LC, LC2, and LC3). This might be due to an interaction between hydroxyl moiety and doped La(OH)_3_ onto the surface of cellulose. For unmodified DEAE cellulose, a prominent peak in the FT-IR spectrum at 1,058 cm^-1^, for C-O stretching band, appeared to shift to a lesser wave number (1,051 cm^-1^) in modified cellulose phases. FT-IR spectra show peaks at 2,897 and 1,152 cm^-1^, corresponding to C-H stretching and bending in all the phases of cellulose. Absorption bands at 557 cm^-1^ observed in only modified cellulose phases were due to La-O stretching vibration as depicted in Figure 4. All these characteristic peaks of DEAE cellulose and La(OH)_3_ detected in the spectrum of nanocomposites confirmed the formation of nanocomposites [40,41].

**Figure 5:**
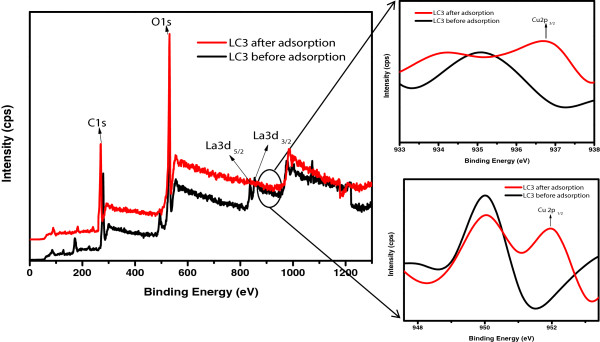
XPS spectra of LC3 phase before and after adsorption of Cu(II).

XPS was performed in order to quantitatively achieve the composition, electronic state, chemical state, and empirical formula of the elements present within a matrix. The XPS spectrum of the material was taken by irradiating it with X-ray beam and obtaining the kinetic energy and number of electrons that escape within 1- to 10-nm region from the material being analyzed. In order to investigate the doping and adsorption phenomena, XPS spectra were taken for best doped cellulose matrix as adsorbent of Cu(II), i.e., LC3, before and after adsorption (Figure 5). For both spectra of LC3 before and after adsorption, the material exhibits peaks at 281.75, 530.96, 834.69, and 851.82 eV, corresponding to C1s, O1s, La3d_5/2_, and La3d_3/2_, respectively. However, new peaks were observed in the LC3 spectrum after adsorption at 936.75 and 951.94 eV, corresponding to Cu2p_3/2_ and Cu2p_1/2_, respectively, as can be depicted in Figure 5. Notably, such peaks of Cu(II) are absent in the spectrum of LC3 before adsorption took place. These characteristic peaks were in agreement with the previous literature [40,42] and further confirmed the formation of doped cellulose matrix with La(OH)_3_ and adsorption of Cu(II).

### Batch method

#### ***Effect of pH***

**Figure 6:**
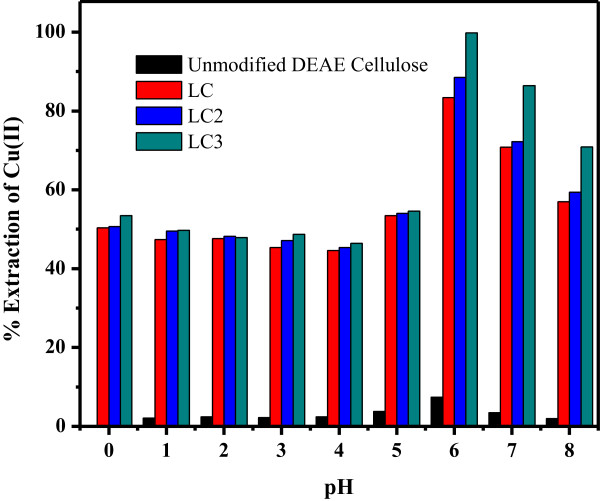
**Effect of pH on the adsorption of 5 mgL**^**-1 **^**Cu(II).** On 25 mg unmodified DEAE cellulose and modified cellulose phases at 25°C.

The effect of pH was investigated for the selective adsorption of Cu(II) ion onto the modified cellulose phases within the range of 1.0 to 8.0 by applying buffer solutions. The percentage extraction of Cu(II) on unmodified DEAE cellulose was 7.47% at optimum pH 6.0 (Figure 6). However, the percentage extractions for LC, LC2, and LC3 were 83.36%, 88.48%, and 99.78%, respectively. Hence, phase LC3 displayed maximum efficiency for the extraction of Cu(II) among all other modified phases. Although the difference of extraction between the unmodified and modified cellulose matrix was enormous, all phases displayed their maximum extraction at pH 6.0 (Figure 6). At lower pH value, new proton acceptor centers are created and may be competed with metal ions adsorption on binding sites of all phases, resulting in the low adsorption of Cu(II) [43]. However, the highest percentage of Cu(II) extraction (99.78%) at pH 6.0 with LC3 phase can be attributed to highest content of negatively charged sites presented on LC3 phase at this specific pH value and as a result of doping the highest content (10%) of La(OH)_3_ on DEAE cellulose surface. These negatively charged sites/centers are able to easily bind with the positively charged Cu(II) ions through electrostatic attraction at pH 6.0. It was noticed that adsorption was further decreased at pH greater than 6.0. This may be ascribed to the precipitation of heavy metal ions above pH 6.0 leading to the reduction of the metal ions in the solution [44].

#### ***Adsorption capacity***

**Figure 7:**
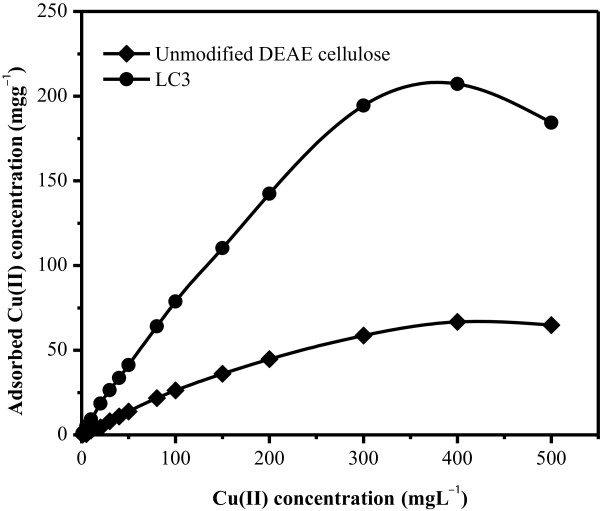
Adsorption performance of Cu(II) onto the surface of unmodified DEAE cellulose and LC3 phases.

Adsorption capacity of Cu(II) on LC3 was estimated by varying amounts (0 to 500 mgL^-1^) of Cu(II) and individually mixing them with 25.0 mg LC3 at pH 6.0 under batch procedure. Uptake capacities of unmodified DEAE cellulose and LC3 for Cu(II) were determined by plotting a breakthrough curve between concentration (mgL^-1^) of Cu(II) versus the milligram of Cu(II) adsorbed per gram of phase (Figure 7). From the adsorption isotherm study, the adsorption capacity of LC3 for Cu(II) was determined to be 207.17 mgg^-1^ which is higher than those previously reported for Cu(II) in other studies (5.2 [45], 12.0 [46], 66.7 [47], 73.5 [48], 110.5 [49], and 160.1 mgg^-1^ [50]). For comparison with the adsorption capacity of LC3 toward Cu(II), the Cu(II) adsorption capacity on the unmodified DEAE cellulose was evaluated and estimated to be 66.67 mgg^-1^ under the same batch conditions as well as LC3, as displayed in Figure 7. This provides that the adsorption capacity of Cu(II) was improved by 211% with the LC3 nanocomposites as compared to that with the unmodified DEAE cellulose.

#### ***Effect of shaking time***

**Figure 8:**
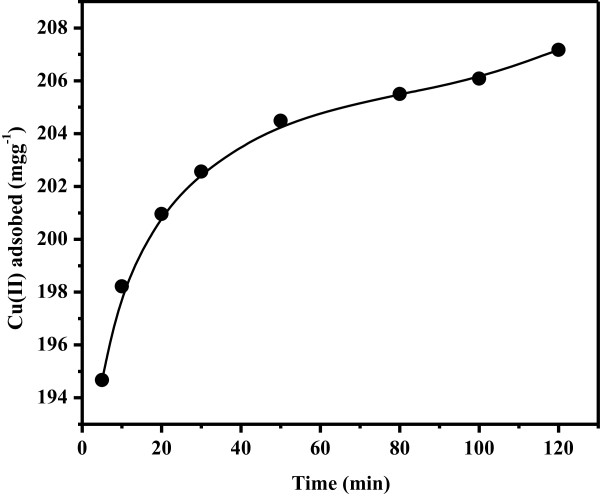
Effect of contact time on Cu(II) adsorption on 25.0 mg LC3 at pH 6.0 and 25°C.

The shaking time effect is considered to be a principal factor when the static technique is exercised for the determination of adsorption capacity values of the metal ion. In this report, the shaking times within 5 to 120 min were investigated to attain the effect of contact time on Cu(II) adsorption. Results indicated fast equilibrium kinetics of Cu(II) adsorption on the surface of LC3 (Figure 8). It was found that over 200 mgg^**-**1^ Cu(II) was adsorbed onto the surface of LC3 within 20 min. However, after 20 min, minor change in the adsorption of Cu(II) was observed, and the value rose up to the maximum value of 207.17 mgg^**-**1^ after 120 min. The uptake capacity of Cu(II) was also raised up to more than 204 mgg^-1^ after 50 min until the maximum adsorption of LC3 toward Cu(II) was reached after 120 min.

#### ***Effect of temperature***

The effect of temperature on the adsorption of LC3 for Cu(II) was studied in order to determine thermodynamic parameters. In this study, the effect of temperature on the adsorption of 25.0 mg LC3 along with 25.0 mL of 5.0 mgL^-1^ Cu(II) solution was investigated at different temperatures, ranging from 298 to 338 K. The results showed that adsorption capacity increases with decrease in solution temperature. This elaborates exothermic nature of adsorption process. Thermodynamic parameters linked with adsorption process, such as change in free energy (∆*G°*, kJmol^-1^), entropy (∆*S°*, Jmol^-1^ K^-1^), and enthalpy (∆*H°*, kJmol^-1^), were calculated by employing the following equations:

(1) $$K_{d}=\left(C_{o}–\;C_{e}/\;C_{e}\right)\times \left(V/m\right)$$

(2) $$\ln\;K_{d}=\Delta \mathrm{S{}^{\circ}}/R–\left(\Delta \mathrm{H{}^{\circ}}/\mathrm{RT}\right)$$

(3) $$\Delta \mathrm{G{}^{\circ}}=-\mathrm{RT}\ln K_{d}$$

where *K*_d_ is distribution adsorption coefficient, *C*_o_ and *C*_e_ denote initial and final concentrations of the metal before and after adsorption, respectively, *V* corresponds to the volume (mL), *m* represents the weight of the phase (*g*), *R* is the universal gas constant (8.314 Jmol^-1^ K^-1^), and *T* corresponds to the temperature in Kelvin.

**Figure 9:**
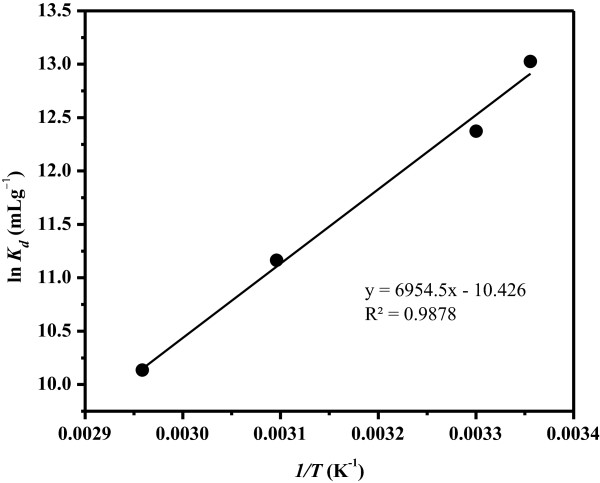
Adsorption isotherm study of Cu(II) extraction by LC3 at different temperatures.

Thermodynamic parameters of ∆*H°* and ∆*S°* were calculated using Equation 2 from the slope and intercept of the linear variation of ln *K*_d_ with the reciprocal of the temperature (*1*/*T*), as displayed in Figure 9 and Table 1. The standard Gibbs free energy change, ∆*G*, was obtained from Equation 3 (Table 1). As can be depicted from Table 1, calculated thermodynamic parameters were all negative. The observed negative ∆*H°* value (-57.82 kJmol^-1^) supports that the adsorption process of LC3 toward Cu(II) is exothermic in nature. The negative ∆*S°* value (-86.68 Jmol^-1^ K^-1^) provides that the system randomness decreases during the adsorption process on the adsorbent surface, a general observation in most of the processes of metal ion uptake [49,50]. Finally, negative values of ∆*G°* suggest that the adsorption mechanism of LC3 toward Cu(II) is a generally spontaneous process and thermodynamically favorable.

**Table 1:** Thermodynamic parameters associated with the adsorption of Cu(II) on LC3 phase

**∆** ***H° *** **(kJmol**^**-1**^**)**	**∆** ***S° *** **(Jmol**^**-1**^ **K**^**-1**^**)**	**∆** ***G° *** **(kJmol**^**-1**^**)**			
		***T*** **= 298 K**	***T*** **= 303 K**	***T*** **= 323 K**	***T*** **= 338 K**
-57.82	-86.68	-32.27	-31.17	-29.98	-28.48

#### ***Adsorption isotherm models***

The adsorption isotherm analysis is very crucial to obtain an equation that accurately corresponds to the results. The obtained adsorption isotherm data for Cu(II) is therefore assessed by two well-known adsorption isotherm models, Langmuir and Freundlich. These two models were utilized to match the equilibrium data and acquire the correlation coefficient (*R*^2^) values to assess fitting parameters. The Langmuir model describes a monolayer adsorption phenomenon onto a completely homogeneous surface with equally available identical adsorption sites along with negligible interaction of adsorbed species. It can be defined by the following equation [51]:

(4) $$C_{e}/q_{e}=\left(C_{e}/Q_{o}\right)+1/Q_{o}b$$

where *C*_e_ is the concentration of un-retained metal ion remaining in the filtrate at equilibrium (mg mL^-1^) and *q*_e_ corresponds to the amount of adsorbed metal ion on adsorbent (mgg^-1^). The symbols *Q*_o_ and *b* refer to Langmuir constants related to the maximum adsorption capacity (mgg^-1^) and affinity parameter (Lmg^-1^), respectively. Langmuir constants can be calculated from a linear plot of *C*_e_/*q*_e_ against *C*_e_ with a slope and intercept equal to 1/*Q*_o_ and 1/*Q*_o_*b*, respectively. Moreover, a dimensionless constant separation factor or equilibrium parameter *R*_L_ is an additional characteristic of the Langmuir model, which is defined by the given equation below:

(5) $$R_{L}=1/\left(1\;+\;bC_{o}\right)$$

where *b* is the Langmuir constant, indicating the nature of adsorption and the shape of the isotherm and *C*_o_ is the initial concentration of the analyte of interest. The *R*_L_ values specify the type of isotherm, and *R*_L_ values within 0 to 1 suggest a favorable adsorption [52].

On the other hand, Freundlich isotherm model is based on the assumption of a reversible adsorption process at multilayers on heterogeneous surface of the phase. It can be expressed by the following equation:

(6) $$\log q_{e}=\log K_{f}+1/n\;\log C_{e}$$

where *K*_f_ and *n* are the Freundlich isotherm constants related to adsorption capacity and intensity of adsorption, respectively. Freundlich constants (*K*_f_ and *n*) can be calculated from the intercept and slope, respectively, of the linear plot of log*q*_e_ versus log*C*_e_.

**Figure 10:**
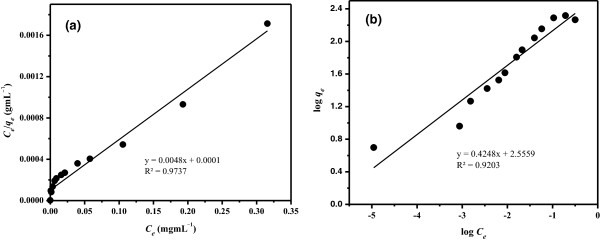
Langmuir (a) and Freundlich (b) adsorption isotherm models for Cu(II) adsorption on LC3 phase at 25°C.

By employing least square fit method, linear plots of Langmuir and Freundlich isotherm models were obtained (Figure 10). Table 2 represents values acquired from these models. From Table 2, it can be seen clearly that Langmuir model is better fitted as compare to Freundlich model. Langmuir model has higher correlation factor (*R*^2^ *=* 0.9737) and close *Q*_0_ value (207.46 mgg^-1^) to that (207.17 mgg^-1^) experimentally obtained from adsorption isotherm study. However, the correlation factor obtained from Freundlich model is less (0.9203) than that obtained from Langmuir model. In addition, the *K*_f_ value 359.66 mgg^-1^ represents large deviation from that obtained from the adsorption isotherm study. Considering these results, it can be concluded that a monolayer adsorption took place on the surface of adsorbent with homogenous adsorption sites. The *R*_L_ value of Cu(II) adsorption on the LC3 is 0.06, validating a favorable adsorption process based on the Langmuir model.

**Table 2:** Langmuir and Freundlich adsorption isotherm parameters for the adsorption of Cu(II) on LC3 phase

**Langmuir model**			**Freundlich model**		
***Q***_**0 **_**(mgg**^**-1**^**)**	***b *** **(Lmg**^**-1**^**)**	***R***^**2**^	***K***_**f **_**(mgg**^**-1**^**)**	**1/*n***	***R***^**2**^
207.46	0.04	0.977	359.66	0.42	0.9203

#### Kinetic models

**Figure 11:**
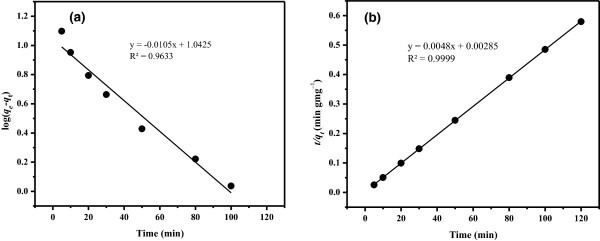
Pseudo- (a) first- and (b) second-order kinetic models of Cu(II) uptake on 25.0 mg LC3 phase at pH 6.0 and 25°C.

Different kinetic models were studied in order to explore inherent kinetic adsorption parameters. These models are applied in order to check fitness of the experimental data, where correlation coefficient (*R*^2^) value is taken as the measure of agreement between the experimental data. In this study, two kinetic adsorption models were evaluated. The equation for pseudo-first-order adsorption kinetic can be expressed as follows:

(7) $$\log\left(q_{e}–q_{t}\right)=\log q_{e}–\left(k_{1}/2.303\right)t$$

where *q*_e_ (mgg^-1^) and *q*_t_ (mgg^-1^) are the amounts of adsorption at equilibrium and at time *t* (min), respectively, and *k*_1_ denotes the adsorption rate constant of pseudo-first-order adsorption (min^-1^). The adsorption rate constant *k*_1_ and the adsorption capacity *q*_e_ were calculated from the slope and intercept of the plot of log*(q*_e_*– q*_t_*)* against *t* and found to be 2.42 × 10^-2^ min^-1^ and 11.03 mgg^-1^, respectively, as illustrated in Figure 11a. In addition, the kinetic equation for pseudo-second-order adsorption can be written as follows:

(8) $$t/q_{t}=1/\upsilon_{o}+\left(1/q_{e}\right)t$$

where *υ*_o_ = *k*_2_ is the initial adsorption rate (mgg^-1^ min^-1^) where *k*_2_ (gmg^-1^ min^-1^) corresponds to the rate constant of the pseudo-second-order adsorption, *q*_e_ (mgg^-1^) is the amount of metal ion adsorbed at equilibrium, and *q*_t_ (mgg^-1^) refers to the amount of metal ion on the adsorbent surface at any time *t* (min). The plot of *t*/*q*_t_ versus *t* was developed in order to deduce kinetic parameters of *υ*_o_ and *q*_e_ from the intercept and slope, respectively.

The correlation coefficient factor (*R*^2^) obtained from pseudo-second-order plot was found to be 0.9999 (Figure 11b). Kinetic parameters *υ*_o_ and *q*_e_ were found to be 350.51 mgg^-1^ min^-1^ and 207.58 mgg^-1^, respectively, and *k*_2_ was estimated to be 0.01 gmg^-1^ min^-1^ for LC3 adsorption for Cu(II) (Table 3). It can be noticeably observed that the value of *q*_e_ obtained from the pseudo-second-order kinetic model is consistent with the outcome of adsorption isotherms, strongly supporting the validity of pseudo-second-order kinetic model.

**Table 3:** Pseudo-first- and second-order kinetic model parameters for extraction of Cu(II) by LC3 phase

***C***_**0 **_**(mg/L)**	***q***_**e,**__**exp **_**(mg/g)**	**Pseudo-first-order kinetic model**			**Pseudo-second-order kinetic model**		
		***k***_**1 **_**(min**^**-1**^**)**	***q***_**e,**__**cal **_**(mgg**^**-1**^**)**	***R***^**2**^	***k***_**2 **_**(gmg**^**-1**^ **min**^**-1**^**)**	***q***_**e,**__**cal **_**(mgg**^**-1**^**)**	***R***^**2**^
400	207.17	2.42E-02	11.03	0.9633	0.01	207.58	0.999

### Performance of proposed method

#### ***Selectivity studies***

Selectivity studies of modified and unmodified DEAE cellulose adsorbents toward different metal ions, including Cd(II), Co(II), Cu(II),Cr(III), Cr(VI), Fe(III), Ni(II), and Zn(II), were investigated by determining the distribution coefficient of all the phases at optimum pH value (pH 6.0). Distribution coefficient (*K*_d_) values were determined from Equation 1.

**Figure 12:**
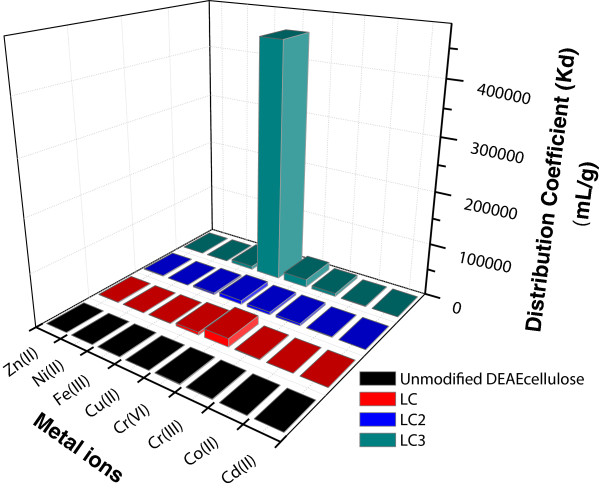
**Selectivity study of 25 mg LC3 phase of different metal ions.** With unmodified DEAE cellulose and modified cellulose phases at pH 6.0 and 25°C.

The values of distribution coefficient along with uptake capacities for all metal ions against different phases of cellulose at optimum pH 6.0 are summarized in Table 4. It can be clearly observed from Table 4 and Figure 12 that the Cu(II) adsorption on LC3 phase has the highest distribution coefficient value (4.54 × 10^5^ mLg^-1^). These results suggested that Cu(II) had the highest selectivity among all the metal ions and specifically toward the LC3 phase. However, the minimum overall value of *K*_d_ is observed in case of Cu(II) adsorption on the unmodified DEAE cellulose. Consequently, the modified phase LC3 provided the best selectivity for the separation of Cu(II) in this study.

**Table 4:** Uptake capacities and distribution coefficient values of different metal ions against unmodified DEAE cellulose and modified cellulose phases at pH 6.0 and 25°C

**Metal ions**	**Unmodified DEAE cellulose**		**LC**		**LC2**		**LC3**	
	***q***_**e **_**(mgg**^**-1**^**)**	***K***_**d **_**(mLg**^**-1**^**)**	***q***_**e **_**(mgg**^**-1**^**)**	***K***_**d **_**(mLg**^**-1**^**)**	***q***_**e **_**(mgg**^**-1**^**)**	***K***_**d **_**(mLg**^**-1**^**)**	***q***_**e **_**(mgg**^**-1**^**)**	***K***_**d **_**(mLg**^**-1**^**)**
Cd(II)	0.62	1.42 × 10^2^	0.75	1.76 × 10^2^	0.00	0.00	0.92	2.24 × 10^2^
Co(II)	0.73	1.71 × 10^2^	0.73	1.70 × 10^2^	1.90	6.15 × 10^2^	1.86	5.93 × 10^2^
Cr(III)	2.33	8.70 × 10^2^	2.15	7.53 × 10^2^	4.01	4.07 × 10^3^	4.27	5.88 × 10^3^
Cr(VI)	2.46	9.67 × 10^2^	4.71	1.61 × 10^4^	4.14	4.83 × 10^3^	4.69	1.51 × 10^4^
Cu(II)	0.18	3.78 × 10^1^	4.17	5.01 × 10^3^	4.42	7.68 × 10^3^	4.99	4.54 × 10^5^
Fe(III)	2.37	8.98 × 10^2^	2.03	6.85 × 10^2^	3.76	3.02 × 10^3^	4.29	6.02 × 10^3^
Ni(II)	0.26	5.46 × 10^1^	0.27	5.69 × 10^1^	0.00	0.00	0.48	1.05 × 10^2^
Zn(II)	0.81	1.92 × 10^2^	0.84	2.03 × 10^2^	2.19	7.79 × 10^2^	1.09	2.79 × 10^2^

#### ***Application in real environmental samples***

The proposed method was applied to real environmental water samples for the extraction of Cu(II) using LC3 phase as an adsorbent. Four different water samples, including tap water, ground water, seawater, and wastewater, were collected from Jeddah, Saudi Arabia. These samples were spiked with Cu(II) and utilized for adsorption experiments with LC3 phase. These samples were studied under the same batch conditions at optimum pH 6.0. The percentage extraction of Cu(II) from these real samples was calculated and reported in Table 5. The results showed that the extraction of spiked Cu(II) water samples using LC3 phase in the range of 89.87% to 98.23%. Thus, these results were satisfactory, and the proposed method is reliable, feasible, and applicable to real sample analysis.

**Table 5:** Determination of Cu(II) at different concentrations in real water samples utilizing LC3 phase

**Environmental samples**	**Added (mgL**^**-1**^**)**	**Un-adsorbed (mgL**^**-1**^**)**	**Percentage extraction**
Tap water	2.0	0.05	97.29
	10.0	0.47	95.26
	50.0	4.35	91.31
Ground water	2.0	0.04	98.23
	10.0	0.40	95.99
	50.0	3.86	92.27
Seawater	2.0	0.06	97.05
	10.0	0.67	93.26
	50.0	4.45	91.11
Wastewater	2.0	0.09	95.73
	10.0	0.67	93.26
	50.0	5.06	89.87

## Conclusions

**Figure 13:**
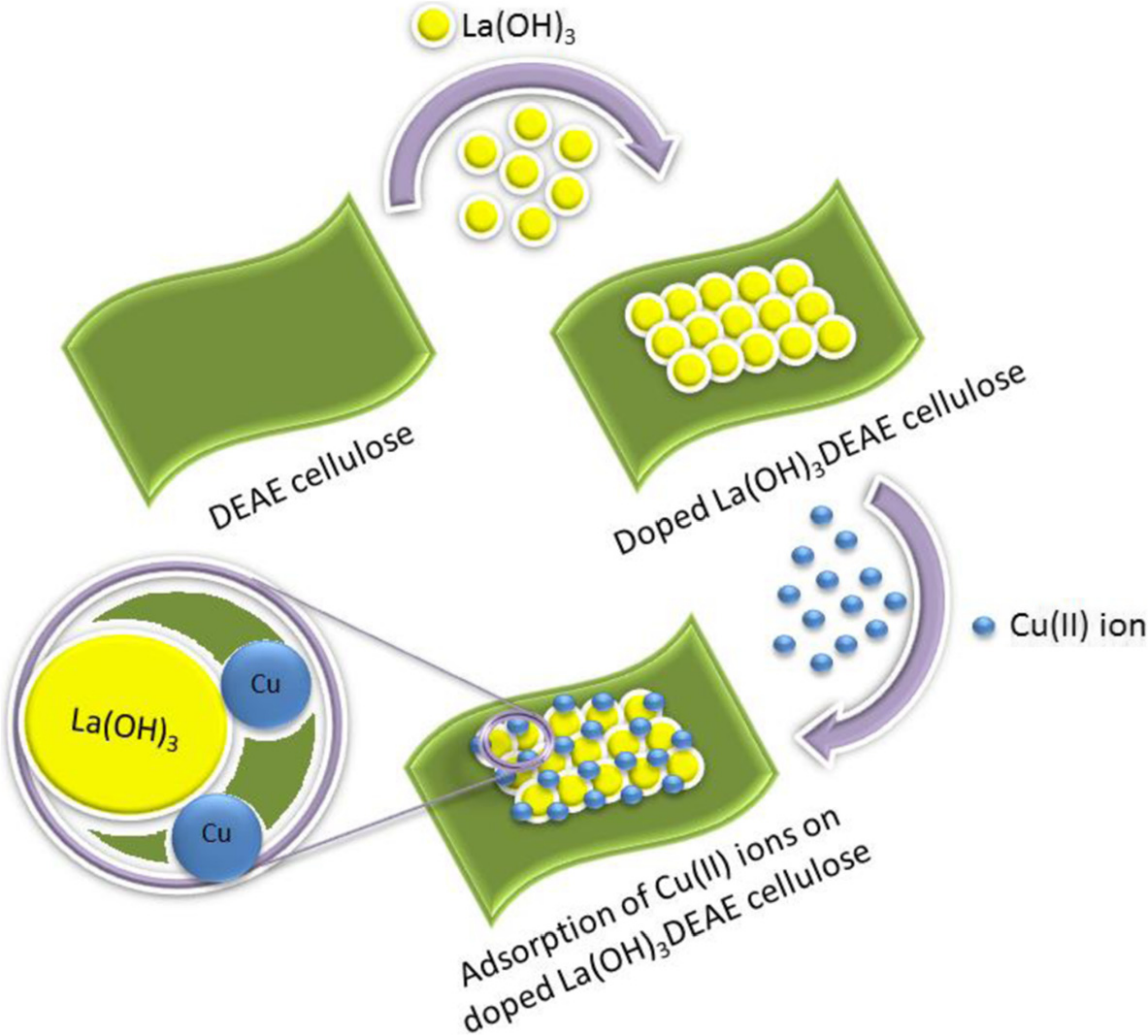
Schematic view of adsorption phenomena on the modified DEAE cellulose adsorbent.

Nanocomposites of surface modified cellulose matrix were prepared successfully by growing different percentages of La(OH)_3_ on DEAE cellulose surface. The morphology of these nanocomposites was confirmed by FE-SEM, EDS, XRD, FT-IR, and XPS. The analytical potential of newly synthesized nanocomposites for selective adsorption and determination of Cu(II) was evaluated. The outcome of the research revealed that among the different percentages of doped La(OH)_3_ phases, the 10% doped La(OH)_3_ phase (LC3) showed significant uptake capacity of Cu(II) in aqueous medium (Figure 13). LC3 phase also presented an excellent sensitivity toward Cu(II) adsorption and achieved good static uptake capacity. About 211% increase in adsorption capacity was achieved by LC3 phase as compared to unmodified DEAE cellulose. Adsorption data were found to be well fitted with the Langmuir isotherm model and followed second-order kinetic. Furthermore, the proposed method was applied to real environmental water samples and achieved adequate results for the extraction of Cu(II). Consequently, the method may display substantial promise for applying it as an applicable approach for a selective separation and detection of Cu(II) in complex matrices.

## Competing interests

The authors declare that they have no competing interests.

## Authors’ contributions

SBK and AMA synthesized the nanomaterials, performed structural analyses of the samples, analyzed the experimental results, and contributed to the manuscript preparation. MUL and HMM coordinated the study, analyzed the data, carried out the metal ion uptake study, and contributed to the manuscript preparation. All authors read and approved the final manuscript.
